# Subcellular localisation of pMEK has a different prognosis in locally advanced head and neck cancer treated with concomitant radiochemotherapy

**DOI:** 10.1186/s12885-016-2869-x

**Published:** 2016-10-28

**Authors:** J. Gomez-Millan, B. Pajares, L. Perez-Villa, A. Carnero, M. Alvarez, V. De Luque, F. Rivas, J. M. Trigo, M. D. Toledo, E. Alba, J. A. Medina

**Affiliations:** 1 Radiation Oncology Department, Hospital Universitario Virgen de la Victoria, Campus Teatinos s/n, 29010 Malaga, Spain; 2 Medical Oncology Department, Hospital Universitario Virgen de la Victoria, Campus Teatinos s/n, 29010 Malaga, Spain; 3 Pathology Department, Hospital Universitario Virgen de la Victoria, Campus Teatinos s/n, 29010 Malaga, Spain; 4 Instituto de Biomedicina de Sevilla (IBIS), Consejo superior de investigaciones científicas, Campus Universitario Virgen del Rocío, Avda, Manuel Siurot s/n, 41013 Sevilla, Spain; 5 Pathology Department, Facultad de Medicina, UMA, Campus Teatinos s/n, 29010 Malaga, Spain; 6 Agencia Sanitaria Costa del Sol, Unidad de Investigación, Autovia A-7, Km 187, 29063 Marbella Málaga, Spain; 7 Red Nacional de Investigación de Servicios de Salud en Enfermedades crónicas (REDISSEC), Madrid, Spain

**Keywords:** Head and neck cancer, Radiochemotherapy, pMEK

## Abstract

**Background:**

MEK1 (MAP2K1) and MEK2 (MAP2K2) are closely related dual-specificity protein kinases which function by phosphorylating both serine/threonine and tyrosine residues of their substrates ERK1 and ERK2, controlling fundamental cellular processes that include cell growth and proliferation. To investigate the prognostic significance of pMEK expression in the nucleus and cytoplasm among patients with locally advanced head and neck cancer treated with concurrent radiochemotherapy.

**Methods:**

Immunohistochemistry was performed on the retrieved archival tissue of 96 patients to detect pMEK, p53 and Ki-67.

**Results:**

Sixty-six percent of patients were positive for pMEK expression in the nucleus and 41 % in cytoplasm. On univariate analysis, high nuclear pMEK was predictive of worse 5y-DFS and 5y-OS, with a trend to significance (26 % vs. 41 %, *p* = 0.09; 36 % vs. 47 %, *p* = 0.07). High cytoplasmic pMEK was predictive of better 5-y OS and 5-y DFS outcomes (61 % vs. 27 %, *p* = 0.01; 46 % vs. 22 %, *p* = 0.02). On multivariate analysis, low cytoplasmic pMEK and high nuclear pMEK predicted worse DFS and OS (*p* = 0.01; *p* = 0.04 and *p* = 0.02; *p* = 0.02 respectively).

**Conclusions:**

Subcellular localisation of pMEK has different prognosis in locally advanced head and neck cancer treated with radiochemotherapy.

## Background

A combination of concurrent radiotherapy and chemotherapy (RCT) is the standard treatment for locally advanced head and neck cancer (LAHNC) [1]. However, despite the intensification of radiotherapy with chemotherapy, the prognosis of these patients is still poor and involves a considerable increase in toxic effects [2]. Thus, in these patients, it is crucial to investigate new molecular targets to improve the therapeutic ratio of treatment with RCT.

Accelerated proliferation is one of the major causes of failure in head and neck cancer treated with radiation and chemotherapy. One mechanism by which tumoural cells increase the proliferation rate in response to fractionated irradiation in head and neck cancer is EGFR phosphorylation and stimulation of the RAS/RAF/MAPK signalling pathway, a key signal transduction pathway of growth factor induced signals [3]. Within this pathway, MEK1 (MAP2K1) and MEK2 (MAP2K2) are closely related dual-specificity protein kinases that are activated by different growth factors and cytokines which function by phosphorylating both serine/threonine and tyrosine residues of their substrates ERK1 and ERK2, controlling fundamental cellular processes that include cell growth and proliferation [4, 5]. There are data that support the overexpression of MEK1 as an independent biomarker of survival in other tumours such as ovarian cancer [6]. However, there are no published investigations that study the value of pMEK as a prognostic factor in head and neck cancer.

In this study, pMEK was selected as a possible prognostic factor among patients treated with RCT. We examined the expression of nuclear and cytoplasmic pMEK in tumour biopsies of LAHNC treated with RCT with regard to their response to RCT, overall survival (OS) and disease free survival (DFS).

## Methods

### Patient data and specimen characteristics

Between March 2000 and December 2010, 105 patients with newly diagnosed locally advanced HNSCC (stage III and IV non-metastatic), who were candidates for radical treatment, received treatment with concurrent radiochemotherapy (RCT). Of the 105 patients, 96 were fully assessable in terms of the availability of pathological specimens. Pretreatment evaluation included physical examination, endoscopy of the upper aerodigestive tract, computed tomography of the neck, and chest X-ray. In the more advanced cases (N3), computed tomography of the chest was performed. Six to eight weeks after treatment, the response was assessed under RECIST criteria. After treatment, patients underwent regular clinical and imaging examinations to assess for the occurrence of HNSCC relapse or death.

Chemotherapy was administered with Cisplatin, treatment for 70 patients was a 100 mg/m^2^ dose every 3 weeks, whereas 26 patients had 40 mg/m^2^ every week. Twenty six patients (27 %) received treatment with conventional fractionation, and the other 70 (73 %) were treated with accelerated fractionation with concomitant boost. Conventional fractionation was administered daily in 2 Gy per fraction, 5 days a week, to 70 Gy in 35 fractions over 7 weeks. Accelerated fractionation with concomitant boost was delivered daily in 1.8 Gy per fraction, 5 days a week, to 54 Gy in 30 fractions over 6 weeks to a clinical target volume encompassing the gross tumour and clinically/radiologically involved nodes along with regions of potential subclinical and microscopic disease. After 32.4 Gy, a second daily fraction of 1.5 Gy (with an interval of at least 6 h) was delivered to a clinical target volume including gross tumour and involved nodes for a total of 1.8 Gy in 12 treatment days. The primary tumour and clinically/radiologically involved nodes received 72 Gy in 42 fractions over 6 weeks, and uninvolved nodes received 54 Gy over 6 weeks. Radiation treatment was planned with three-dimensional conformal radiotherapy. The median dose of radiotherapy was 71.4 Gy (70–74 Gy).

### Immunohistochemistry

Tumour samples were collected during diagnosis. Immunohistochemistry (IHC) was performed on formalin-fixed, paraffin-embedded (FFPE) tissue. Heat-induced antigen retrieval was performed using 0.05 mol/L Tris buffer (pH 10.0) heated to 95 °C in a steamer for 20 min. Qualitative detection was performed as follows: for p53 (SP5, Ready to Use, MasterDiagnostica, Granada, Spain), Ki-67 and p16 (CINtec® Histology Kit (MTM Laboratories AG, Germany), Phospho MEK1/2 (1:100 SER 217/221, Cell Signalling, Darmstadt, Germany). Slides were incubated with primary antibody and stained according to the standard EndVision Flex + (Dako, Copenhagen, Denmark) (K8012). As the chromogen, we used DAB and slides which were counterstained with hematoxylin. Sections of tumour tissue known to express the investigated antigens were used as positive controls. Negative controls were routinely carried out by substituting the primary antibody for non-specific IgG. The control sections were treated in parallel with the samples in the same run.

**Fig. 1:**
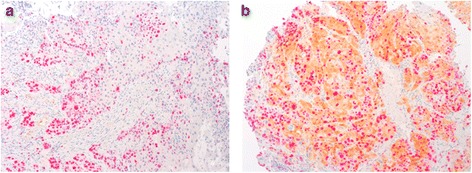
Tumor cells showing negative staining for p16 and positive staining for Ki-67 (**a**). Positive immunostaining for p16 with some cells showing positivity for nuclear staining with Ki-67 (**b**)

**Fig. 2:**
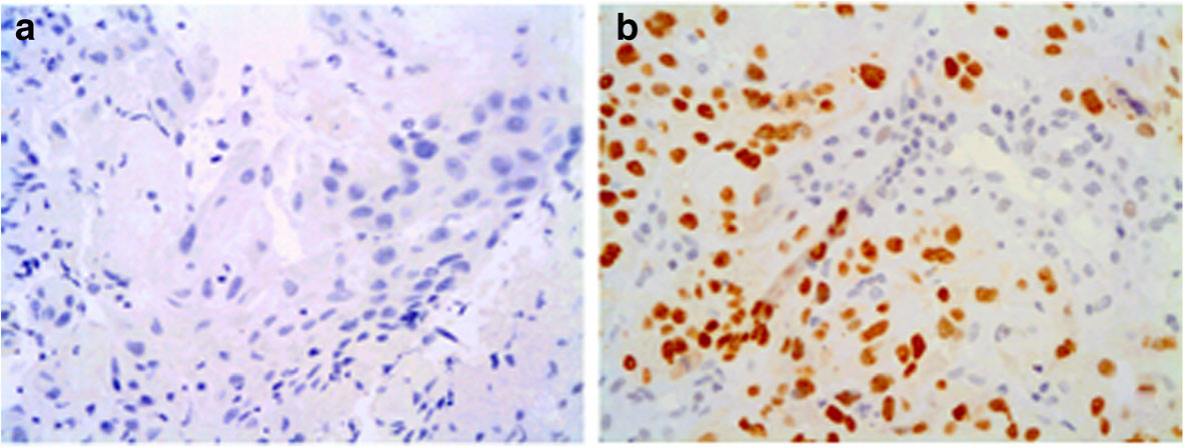
Tumour cells showing negative immunostaining for p53. **a** Positive immunostaining for p53 (**b**)

**Fig. 3:**
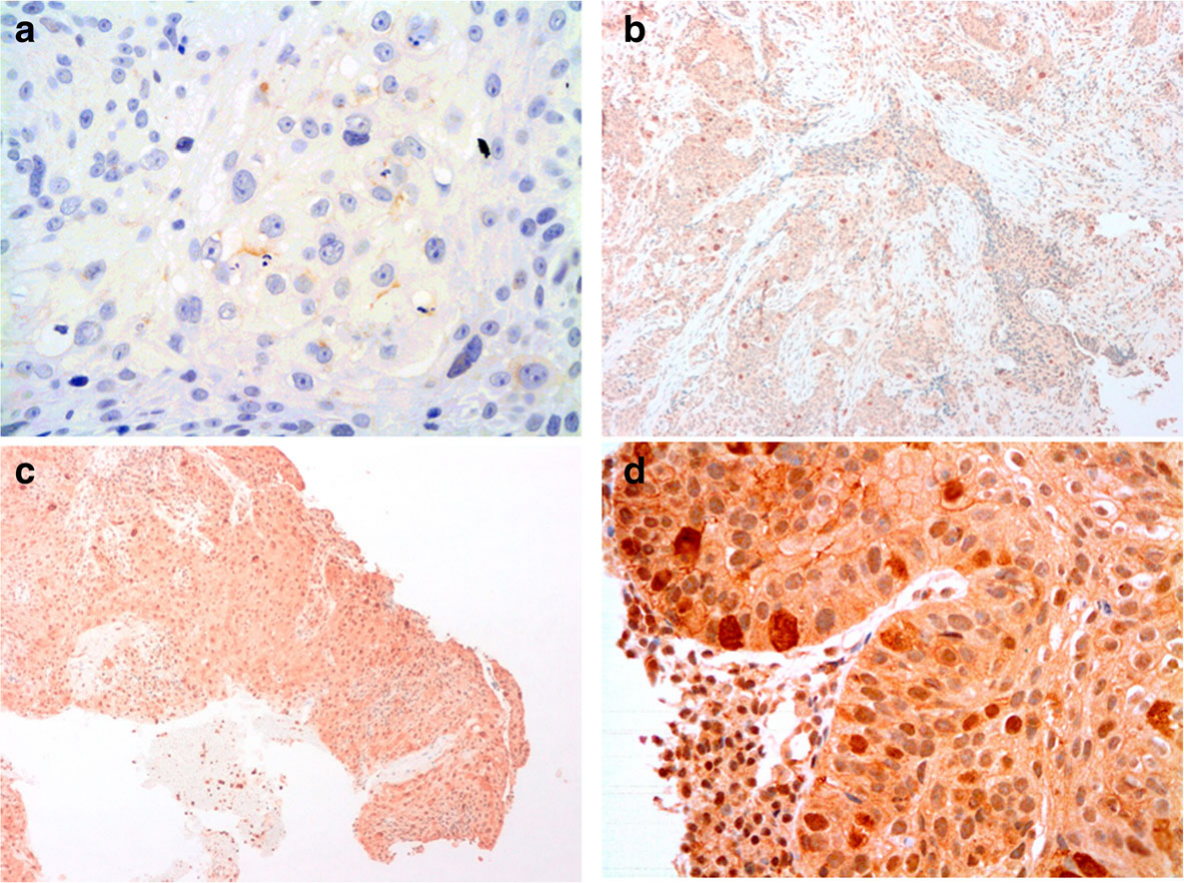
Immunostaining for pMEK. **a** Tumours with absence of pMEK expression (0), **b** low expression (1),**c** moderate expression (2), **d** and high expression (3) in nucleus and cytoplasm

Immunohistochemical staining was evaluated independently by two authors, who were blinded to the clinical data. The ratio between the number of cells that expressed p16, Ki-67 (Fig. 1) and p53 (Fig. 2) and the whole number of tumour cells was calculated. For statistical purposes, accumulation or overexpression of p53 was considered present if >10 % of tumoural cells showed nuclear positivity [7]. Tumours were considered to have a high proliferative index if Ki-67 was positive in >/= 20 % of the cells [8]. Expression of p16 was classified dichotomously as either p16-positive (strong, diffuse staining) or p16 negative [9]. Regarding pMEK, patients were divided considering staining intensity in absence of expression (0), low intensity (+1), moderate intensity (+2) and high intensity (+3). The expression of pMEK was categorised as high expression (average intensity of +2 and +3) and low expression (average intensity of 0 and +1) (Fig. 3). pMEK positivity was considered when at least 5 % of the cells presented a high expression of pMEK (average intensity of +2 and +3).

### Statistical Analysis

The association between clinical and molecular characteristics and prognostic markers was compared using the chi-square test and Fisher’s exact test when appropriate. The end points of interest used in this study were overall survival (OS), disease-free survival (DFS) and tumour relapse. OS was defined by the time that elapsed from first treatment until the event of death due to any cause. DFS was defined by the time that elapsed from the beginning of RCT to documented relapse, or death for any cause. Tumour recurrence was defined as disease recurrence any time after RCT treatment. The pattern of occurrence of the different end points was carried out by estimating Kaplan-Meier survival curves. The threshold for significance for two-side analysis was set to *p* > 0.05. Multivariate survival analysis was conducted using a multivariable Cox regression model. The categorised covariates that showed a trend (*p* < 0.1) in the univariate analysis were put into a back-step multivariate Cox regression analysis. *P*-values <0.05 were considered significant. All analyses were carried out using R and SPSS version 15.0 software.

## Results

### Patient characteristics

**Fig. 4:**
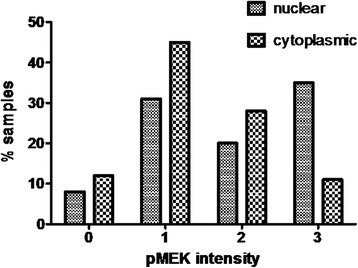
Distribution of tumours with absence of pMEK expression (0), low expression (1), moderate expression (2), and high expression (3) in nucleus and cytoplasm

Of the 96 patients included, the vast majority in both groups were male with ECOG 0-1 performance status and stage IV disease at the time of diagnosis. Fifteen patients (18 % of the total) showed positivity for p16. Of those, two patients (2 % of the total) were smokers. Eighty eight, 84 % of the patients, showed nuclear or cytoplasmic pMEK. Nuclear pMEK staining intensity was absent (0) in eight patients (8 %), weak (1+) in 31 patients (32 %), intermediate (2+) in 21 patients (22 %), and high (3+) in 36 patients (38 %). Cytoplasmic pMEK staining intensity was absent (0) in 12 patients (13 %), weak (1+) in 45 patients (47 %), intermediate (2+) in 29 patients (30 %) and high (3+) in 10 patients (10 %) (Fig. 4). Although pMEK positivity was considered when at least 5 % of the cells presented a high expression of pMEK (average intensity of +2 and +3), 90 % of the tumour samples considered as high expression presented more than 50 % stained cells.

Patients were divided into low pMEK expression (0, 1+) or high expression (2+, 3+) based on the level of staining intensity (Fig. 1). At diagnosis, 66 and 41 % of the patients were categorized as having high pMEK expression in the nucleus and cytoplasm respectively. Patients with a high pMEK in cytoplasm intensity were more likely to present less advanced disease in the neck than patients with a low expression, with 77 % of patients presenting a N0-1 stage in the neck compared with 23 % of patients with N2-3 stage (*p* = 0.02). Characteristics of the patients are shown in Table 1.

**Table 1:** Patients characteristics

Characteristic	Low nuclear pMEK	High nuclear pMEK	*P* value	Low cytoplasmic pMEK	High cytoplasmic pMEK	*P* value
N of patients	33 (34 %)	63 (66 %)		57 (59 %)	39 (41 %)	
Age (y)			0.64			0.65
< 50	6 (18 %)	14 (22 %)		11 (19 %)	9 (23 %)	
>/=50	27 (82 %)	49 (78 %)		46 (81 %)	30 (77 %)	
Sex			0.5			0.01
Male	31 (94 %)	61 (97 %)		57 (100 %)	35 (90 %)	
Female	2 (6 %)	2 (3 %)		0 (0 %)	4 (10 %)	
Current smoker			0.78			0.36
No	2 (6 %)	3 (5 %)		2 (4 %)	3 (8 %)	
Yes	31 (94 %)	60 (95 %)		55 (96 %)	36 (92 %)	
ECOG			0.96			0.71
0–1	31 (94 %)	59 (93 %)		53 (93 %)	37 (95 %)	
2–3	2 (6 %)	4 (6 %)		4 (7 %)	2 (5 %)	
Primary tumor			0.24			0.14
Oropharynx	22 (67 %)	49 (78 %)		39 (68 %)	32 (82 %)	
Other	11 (33 %)	14 (22 %)		18 (32 %)	7 (18 %)	
T classification			0.59			0.32
T1-2	3 (9 %)	8 (13 %)		5 (9 %)	6 (15 %)	
T3-4	30 (90 %)	55 (38 %)		52 (91 %)	33 (85 %)	
N classification			0.29			0.02
N0-1	9 (27 %)	24 (38 %)		26 (46 %)	30 (77 %)	
N2–3	24 (73 %)	39 (62 %)		31 (54 %)	9 (23 %)	
P16			0.45			0.93
Negative	24 (86 %)	43 (80 %)		41 (82 %)	26 (81 %)	
Positive	4 (14 %)	11 (20 %)		9 (18 %)	6 (19 %)	
P53			0.05			0.91
< 10 %	22 (67 %)	29 (46 %)		30 (53 %)	21 (54 %)	
≥ 10 %	11 (33 %)	34 (54 %)		27 (47 %)	18 (46 %)	
Ki-67			0.04			0.34
< 20 %	22 (67 %)	28 (44 %)		32 (56 %)	18 (46 %)	
≥ 20 %	11 (33 %)	35 (56 %)		25 (44 %)	21 (54 %)	

*Abbreviations*: *ECOG* Eastern Cooperative Oncology Group

We analysed the possible association between the site of the primary tumour and pMEK positivity and there was not a significant correlation between pMEK expression and tumour location. Thus, 49 of 71 patients (69 %) with non-oropharyngeal cancer presented nuclear pMEK positivity compared with 14 of 25 patients (56 %) with oropharynx cancer (*p* = 0.24). Moreover, 32 of 71 patients (45 %) with non-oropharyngeal cancer presented cytoplasmic pMEK positivity compared with seven of 25 patients (28 %) with oropharynx cancer (*p* = 0.16).

High nuclear pMEK expression was associated with a higher proliferation rate measured with Ki-67 expression. 56 % of tumours with high nuclear pMEK presented high expression of Ki-67 and 67 % of tumours with low nuclear pMEK presented low expression (*p* = 0.04). Furthermore, high nuclear pMEK expression was significantly associated with expression of p53: 54 % of tumours with high nuclear pMEK presented expression of p53 compared with 33 % in tumours with low nuclear pMEK expression (*p* = 0.05).

We analysed p16+ distribution in patients with less or equal to 50 years compared with less than 50 years old. The rate of p16+ in patients less or equal to 50 years old was 17.6 % (three patients), compared with 18.5 % in patients older than 50 years (*p* = 0.94).

### Subcellular localisation of pMEK localisation expression is associated with outcome

With a median follow up of 48 months, the 5-year OS and DFS were 48 % (median 39 months) and 32 % (median 19 months) respectively. Table 2 lists the univariate analysis for DFS and OS for various prognostic factors. Among the different clinicopathological factors studied, neck stage (N0-1 vs N2-3) was the only factor with prognostic significance, with a 5-year OS of 49 % in patients with less advanced neck disease (N0-1) compared with 27 % in more advanced neck disease (N2-3) (*p* = 0.003), and a 5-year DFS of 46 % in N0-N1 patients compared with 18 % in N2–3 (*p* = 0.004).

**Table 2:** Univariate analysis

	DFS		OS	
Variable	HR (95 % CI)	*P* value	HR (95 % CI)	*P* value
Age (<50 vs ≥50)	1.09 (0.60–1.90)	0.70	1.24 (0.73–2.12)	0.43
Oropharynx vs non-oropharynx	1.30 (0.82–2.29)	0.23	1.20 (0.73–2.10)	0.40
T stage (T1/2 vs T3/4)	0.91 (0.43–1.91)	0.79	1.03 (0.47–2.27)	0.98
N stage (0–1 vs 2–3)	2.03 (1.26–3.28)	0.01	2.11 (1.20–3.50)	0.01
ECOG performance status (0 vs ≥1)	1.50 (0.87–2.47)	0.14	1.50 (0.87–2.58)	0.14
P16	1.30 (0.73–2.51)	0.34	1.36 (0.71–2.59)	0.35
Ki-67	0.82 (0.51–1.33)	0.42	0.95 (0.57–1.56)	0.83
P53	0.99 (0.61–1.60)	0.90	1.10 (0.67–1.82)	0.67
Nuclear pMEK	1.58 (0.93–2.07)	0.09	1.70 (0.95–3.02)	0.07
Cytoplasmic pMEK	0.55 (0.32–0.92)	0.02	0.48 (0.28–0.84)	0.01

*Abbreviations*: *CI* confidence interval; *DFS* disease-free survival; *ECOG* Eastern Cooperative Oncology Group; *HR* hazard ratio; *OS* overall survival

**Fig. 5:**
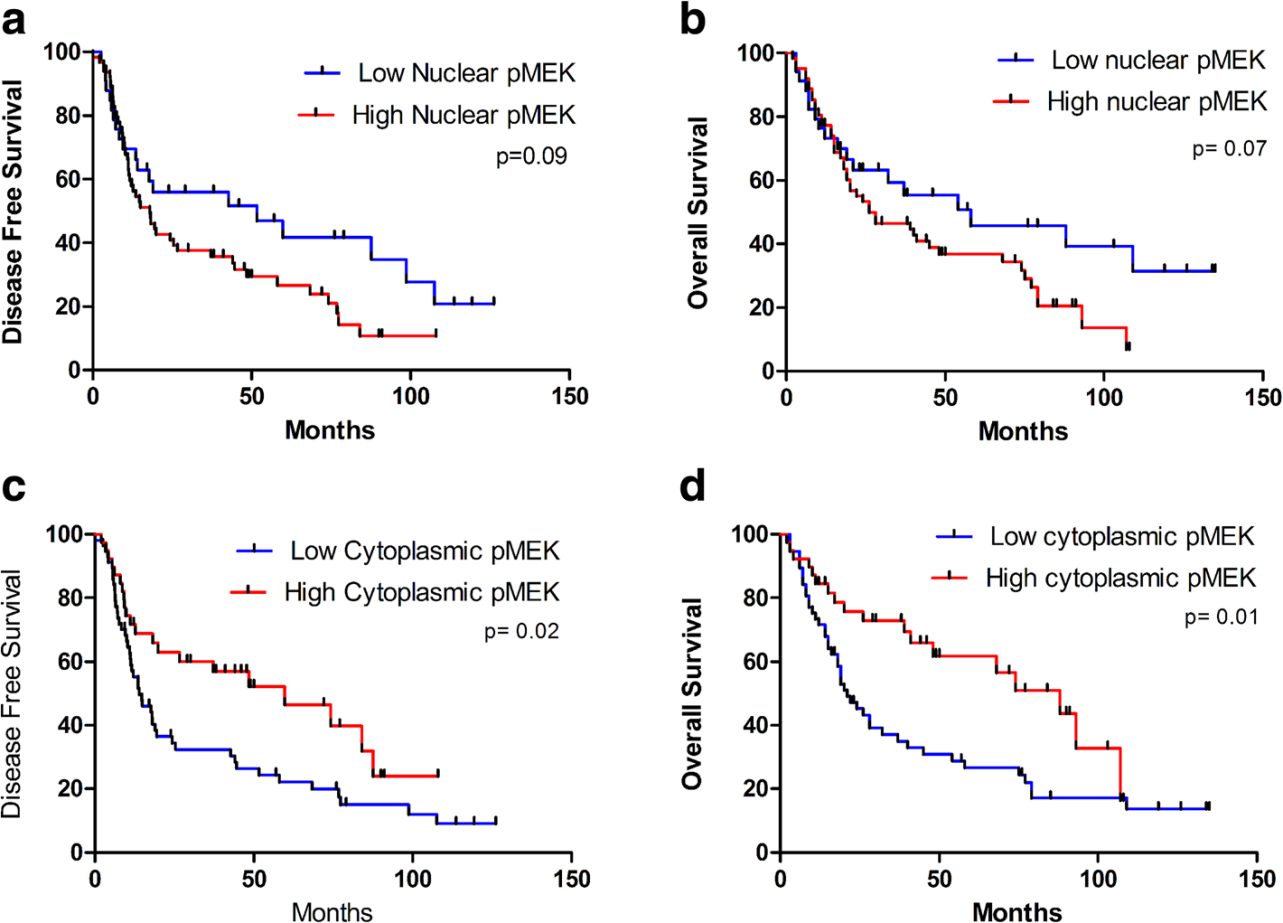
Kaplan-Meier plots for disease-free survival and overall survival according to low versus high expression of pMEK in the nucleus (**a**, **b**) and cytoplasm (**c**, **d**)

To further establish the prognostic significance of pMEK, patients were stratified on the basis of nuclear and cytoplasmic pMEK expression. High nuclear pMEK was predictive of worse DFS and OS, without reaching statistical significance. On the other hand, high cytoplasmic pMEK was predictive of better OS and DFS outcomes (Fig. 5). The 5-year OS for high nuclear pMEK was 36 % compared with 47 % for low nuclear pMEK (HR 1.70; CI 0.95–3.02; *p* = 0.07), and 5-year DFS was 26 % compared with 41 % respectively (HR 1.58; CI 0.93–2.70; *p* = 0.09). Regarding cytoplasmic pMEK, patients with high pMEK showed a 5-year OS of 61 % compared with 27 % when nuclear pMEK was low (HR 0.48; CI 0.28–0.84; *p* = 0.01), and a DFS of 46 % with high pMEK compared with 22 % with low pMEK (HR 0.55; CI 0.32–0.92; *p* = 0.02). On multivariate analysis, after adjustment for T stage, ECOG, and p16, advanced N stage, low cytoplasmic pMEK and high nuclear pMEK predicted worse DFS and OS (Table 3).

**Table 3:** Multivariate analysis

	DFS		OS	
Variable	HR (95 % CI)	*P* value	HR (95 % CI)	*P* value
T stage (T1/2 vs T3/4)	0.82 (0.31–2.17)	0.69	0.94 (0.36–2.46)	0.90
N stage (0–1 vs 2–3)	2.34 (1.33–4.04)	0.03	2.15 (1.25–3.70)	0.06
ECOG performance status (0 vs ≥1)	1.74 (0.93–3.26)	0.08	1.67 (0.93–2.99)	0.09
P16	1.02 (0.53–1.96)	0.95	1.03 (0.54–1.96)	0.92
Nuclear pMEK	2.21 (1.14–4.30)	0.02	2.12 (1.12–3.99)	0.02
Cytoplasmic pMEK	0.48 (0.23–0.85)	0.01	0.53 (0.29–0.97)	0.04

*Abbreviations*: *CI* confidence interval; *DFS* disease-free survival; *ECOG* Eastern Cooperative Oncology Group; *HR* hazard ratio; *OS* overall survival

## Discussion

In this study we analyse the expression of pMEK in the nucleus and cytoplasm in patients with LAHNC treated with RCT. We show that patients with tumours that show moderate to high nuclear pMEK intensity present a lower OS and DFS compared with those who do not. Proliferation is one of the major causes of failure in head and neck cancer treated with radiation and chemotherapy, and the MAPK signal transduction pathway is one of the most important routes for the proliferation of head and neck cancer cells. In the MAPK pathway, MEK1 and MEK2 are the only activators of ERK2, processing inputs from multiple upstream kinases [10]. As a result, ERK1/2 activates different transcription factors and protein kinases, controlling the transcription and translation of genes that promote proliferation. Our results show that high nuclear expression is significantly associated with a higher proliferation rate measured with Ki-67 expression (*p* = 0.04). Although the negative prognostic impact of pMEK has been described in other tumours [6], to our knowledge there are no studies in the literature that investigate the prognostic role of pMEK in head and neck cancer.

Our results show that p16 is not a prognostic factor. The lower p16 positive prevalence in our patients compared with data from different meta-analysis (22–34 % in HNSCC and 30–41 % in OPSCC) [11, 12] confirms the previous results published by our group [9, 13] and is possibly due to the epidemiologic profile of our population, which had a high proportion of heavy tobacco users. In this study, the vast majority of our patients were current smokers (95 %), and only two patients were p16 positive and non-smokers.

Tumour suppressor protein p53 plays a role in the regulation of genes involved in cell cycle and growth arrest, apoptosis and DNA repair, maintaining genomic stability [14, 15]. Mutation of TP53 is one of the most frequently detectable genetic alterations in HNSCC in tumours associated with tobacco and alcohol consumption [16], and this mutation generally results in inhibition of function, limitless proliferation and immortalisation [17]. Our results show that a significantly higher proportion of tumours with high nuclear pMEK presented expression of p53 (54 % in high nuclear pMEK compared with 33 % in low nuclear pMEK, *p* = 0.05). A link between the mutational status of p53 and activation of the Raf/Mek/Erk cascade has recently been reported in preclinical studies, showing that in the presence of Ras oncogenes and an inactive p53/p21 axis, activation of the Raf/Mek/Erk cascade leads to sustained cell proliferation [18, 19].

Moreover, besides regulation of cell cycle progression, the RAF/MEK/ERK signalling pathway may induce cellular responses relevant to cancer survival, such as protection from apoptosis [20, 21]. It is well known that RAF/MEK/ERK can phosphorylate BAD on S112, allowing Bcl-2 to form homodimers, generating an antiapoptotic response [22, 23]. Moreover, the association found between nuclear pMEK and p53 overexpression supports the suggestion that antiapoptotic response may well be an important mechanism of resistance to RCT in head and neck cancer.

Considering the importance that MEKK/1/2 may have in the induction of proliferation, MEK inhibitors might be potentially efficacious for the treatment of head and neck cancer. There has been interest in developing pharmacologic inhibitors of MEK as a means to blocking ERK activation in tumours with activating mutations of MEK1 or MEK2 such as ovarian, melanoma, colorectal and lung cancer [10]. The MEK inhibitor trametinib has been shown to increase overall survival in patients with BRAFV600-mutated melanoma [24] and has recently been approved for use in metastatic melanoma. In head and neck cancer, accelerated proliferation in response to ionising radiation through the activation of proliferative signalling pathways might be mitigated with MEK inhibitors. Trametinib is under investigation in combination with AKT inhibition in solid tumours including HNSCC (NCT01725100).

Secondly, we have identified cytoplasmic pMEK as a promising favourable prognostic factor for OS and DFS. Patients with cytoplasmic pMEK present superior OS and DFS compared with patients who do not express pMEK. Although pMEK rapidly translocates to the nucleus to exert its action on proliferation, it has been described that it remains in the nucleus for a short period of time due to a rapid export by the NE/Exportin system, giving rise to an apparent cytoplasmic localisation [25, 26] that predominates in resting cells [27]. Thus, although the functions of MEKK shuttling and cytoplasmic MEK are not fully understood [28], it might be hypothesised that the subcellular localisation of pMEK may determine different functions and may have different prognostic implications, with poor survival and an increase in proliferation markers in nuclear pMEK compared with better survival and decrease in proliferation markers in cytoplasmic pMEK. Although these finding must be confirmed, cytoplasmic pMEK may identify a subgroup of patients with a good prognosis for whom deintensification therapies might be investigated.

## Conclusions

The expression of pMEK is a prognostic factor in LAHNC treated with CRT. Tumours with nuclear pMEK expression present a higher proliferation rate, showing unfavourable survival, whereas tumours with cytoplasmic expression of pMEK present a lower proliferation rate and favourable survival. Investigation of MEK inhibitors is needed to increase the prognosis of LAHNCs that are frequently not controlled with biologically non-specific treatments such as RCT. Finally, cytoplasmic pMEK expression should be explored as a prognostic biomarker in head and neck cancer.

## Abbreviations

DFS: Disease free survival
ECOG: Eastern Cooperative Oncology Group
HR: Hazard ratio
LAHNC: Locally advanced head and neck cancer
OS: Overall survival
RCT: Concurrent radiotherapy and chemotherapy
